# ATP Can Act as a Stabilizer on Neutral Macromolecules

**DOI:** 10.1021/acs.jpclett.5c02467

**Published:** 2025-10-07

**Authors:** Cansin Ayvaz, Yaren S. Ozdogan, Dilsad S. Peker, Aykut Erbaş, Halil I. Okur

**Affiliations:** † Department of Chemistry, Faculty of Science, 52948 Bilkent University, 06800 Ankara, Turkey; ‡ National Nanotechnology Research Center (UNAM), 52948 Bilkent University, 06800 Ankara, Turkey

## Abstract

Adenosine triphosphate (ATP), an important biomolecule, plays a vital role in delivering cellular energy for various bioprocesses. It was recently shown that ATP also serves as a hydrotrope, destabilizing protein coacervates. Herein, we studied the influence of ATP and relevant small molecules (adenine, adenosine, adenosine monophosphate (AMP), and triphosphate (TP)) on the phase transition of macromolecules, i.e., poly­(*N*-isopropylacrylamide), to explore the underlying mechanism of hydrotropic action of ATP. A multi-instrumental approach, utilizing the Lower Critical Solution Temperature (LCST), Hydrogen-Nuclear Magnetic Resonance (^1^H NMR), and ATR Fourier-Transform Infrared (ATR-FTIR), solvation shell spectroscopy, along with all-atom molecular dynamics simulations were adopted. Adenine and adenosine show a negligible effect on the solubility of macromolecules, whereas ATP, AMP, and triphosphate exhibited dominant salting-out behavior, and promoted the aggregation of neutral macromolecules. ATR-FTIR measurements support the salting-out behavior at physiological ATP concentrations (<0.1 M). In line with this, no apparent evidence for specific binding interaction between the macromolecule and ATP was observed in spectroscopic measurements, as well as MD simulations. At elevated concentrations, ATP self-associates into small clusters, resulting in the destabilization of the PNIPAM chain in its collapsed state. Overall, we demonstrate that only the presence of disordered neutral macromolecules, rich in valine-like pendant isopropyl group, are not sufficient for effective hydrotropic action of ATP; rather, ATP can stabilize such macromolecules with an excluded volume effect at physiological concentrations.

## Graphical abstract

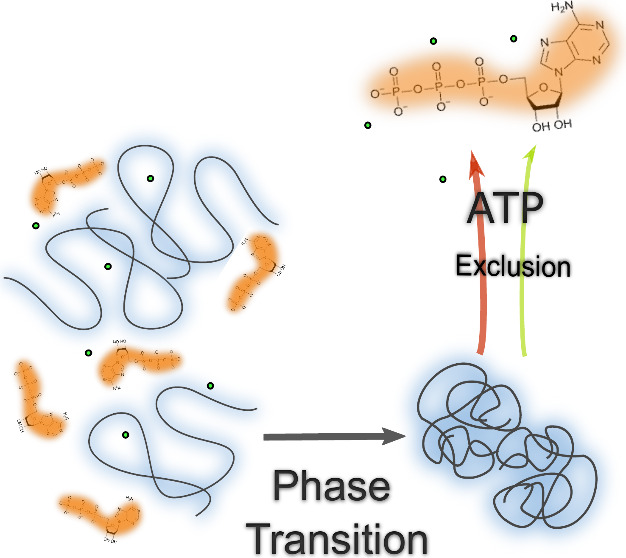

Adenosine triphosphate (ATP) serves as the energy currency of cells, and has been recently discovered to also functioning as a biological hydrotrope.^1^ A hydrotrope is a small molecule that enhances the solubility of water-insoluble or more hydrophobic substances. ATP has a structure known as nucleoside triphosphate, consisting of adenine, ribose sugar, and triphosphate groups. It exhibits an amphiphilic nature due to the presence of both charged, hydrophilic group (triphosphate) and less hydrophilic residues (Figure 1a). This unique property is relevant to its biological roles, including its solubility-enhancing function. In general, cells maintain ATP concentrations in the sub-10 mM (mM) range.^2,3^ However, ATP levels can reach much higher in specific cellular compartments, such as 0.1 M within the chromaffin granules of the adrenal medulla.^1,4–6^

**Figure 1:**
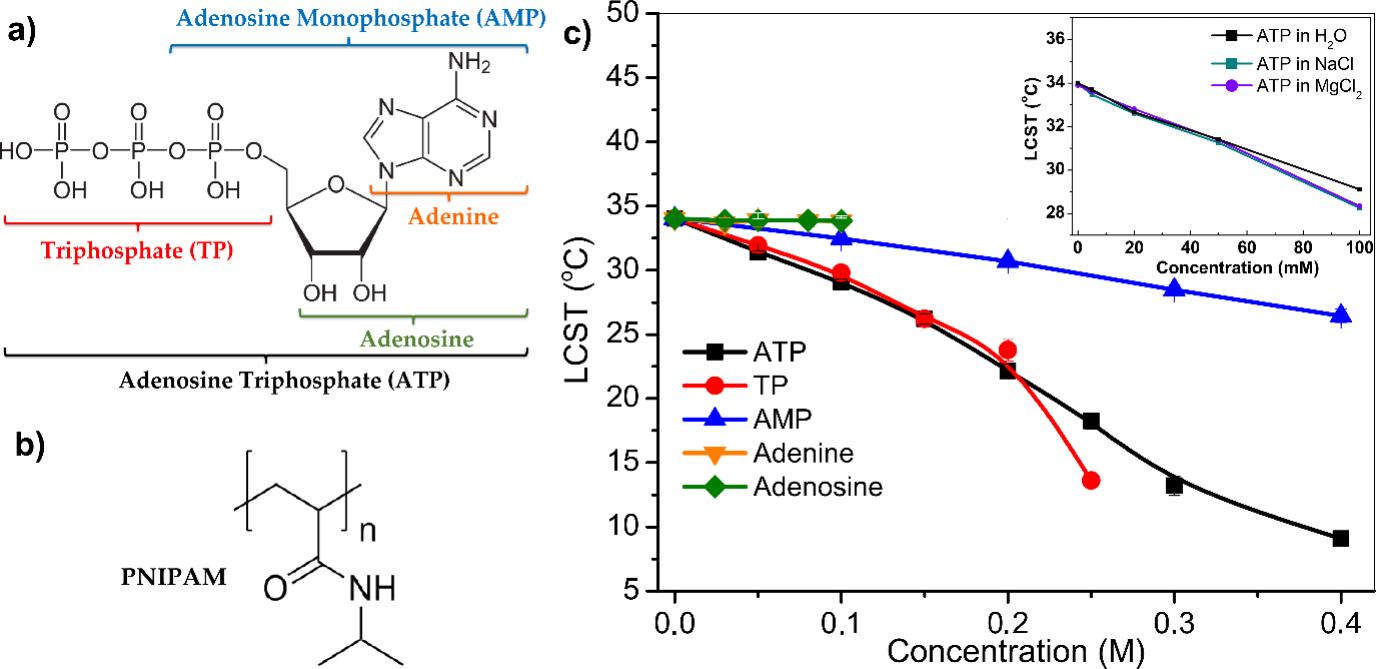
a) The structures of adenosine triphosphate (ATP, black), triphosphate (TP, red), adenosine monophosphate (AMP, blue), adenine (orange), and adenosine (green) are shown. Counter cations are not shown for simplicity. b) The molecular structure of poly­(*N*–isopropylacrylamide) (PNIPAM). c) LCST of 5 mg/mL PNIPAM as a function of ATP, TP, AMP, Adenine, and Adenosine concentrations (in molarity). The inset shows the LCST of 5 mg/mL PNIPAM in the presence of ATP in MgCl_2_, ATP in NaCl, and ATP in aqueous solutions as a function of ATP concentrations (mM). Additional salt was kept at 10 mM for NaCl and MgCl_2_. Error bars indicate the standard deviation of data and are within the data points for many data points. The solid lines are a guide to the eye.

In recent years, the mechanistic study of interactions between osmolytes and macromolecules has gained significant attention, but it remained limited to mostly common osmolytes, such as urea and TMAO.^7–10^ The surprising finding by Patel et al. suggests an additional role of ATP in enhancing the solubility of proteins, preventing protein aggregation, and destabilizing liquid–liquid phase separation (LLPS) of intrinsically disordered proteins, including FUS, TAF15, hnRNPA3, and PGL-3, in biological systems.^1^ However, opposing results were also shown for globular proteins, with attributing the enhanced stability of proteins that is caused by not a classical hydrotropic effect but a Hofmeister effect.^11^ These experimental studies over various macromolecules suggest that ATP’s influence on proteins should be complex. Other attributions to ATP further include the inhibition of fibrillation in intrinsically disordered proteins from unique binding properties, charge–charge interactions that are distinct from either hydrotropic or Hofmeister effects.^11^

The term hydrotrope was first introduced by Neuberg in 1916,^12^ and later, McKee described this phenomenon as a salting-in process, which refers to the enhancement of the solubility of hydrophobic compounds in water, facilitated by the presence of certain ions or small molecules.^13^ After the hydrotropic effect of ATP has been introduced,^1^ its ability to enhance solubility and inhibit fibrillation of intrinsically disordered proteins (IDPs), which lack a well-defined three-dimensional structure, has been strengthened by numerous experimental and simulation-based observations.^14–25^ This phenomenon is attributed to ATP’s salting-in influence. Note that all tested proteins have at least charged surface groups, if not specific ATP binding sites, that can facilitate ATP macromolecule interactions. Conversely, it has also been demonstrated that ATP can increase the stability of folded proteins that adopt stable three-dimensional configurations, and this can be associated with ATP’s salting-out behavior as a strongly hydrated anion.^11,17,26–30^ Thus, the intricate mechanism underlying ATP’s action on various macromolecules is not fully understood. In this work, we explored the interaction of ATP and relevant small molecules (Figure 1a) with neutral macromolecules and investigated ATP’s salting-in, salting-out actions and its hydrotropic nature. To achieve this, a multi-instrumental method, including lower critical solution temperature (LCST, aka phase transition temperature) of macromolecules, nuclear magnetic resonance (NMR), and ATR Fourier-transform infrared spectroscopy (ATR-FTIR), coupled with molecular dynamics (MD) simulations was adopted. Model macromolecule poly­(*N*–isopropylacrylamide) (PNIPAM) (Figure 1b) that contains a valine-like pendant isopropyl structure and displays a random-coil-like configuration akin to intrinsically disordered proteins were employed. This macromolecule can undergo a coil-to-globule transition at the phase transition temperature (Figure S1).

Figure 1c shows the phase transition measurements of PNIPAM solutions as a function of adenosine triphosphate (ATP), triphosphate (TP), adenosine monophosphate (AMP), adenine, and adenosine concentrations (see the structures in Figure 1a). First, PNIPAM has a phase transition temperature (LCST) of 34.02 °C with no additives (in water). The LCST values of PNIPAM in the presence of both adenine and adenosine (green circles and orange triangles) remained nearly unchanged, indicating that these relatively hydrophobic groups of ATP do not have a significant effect on the phase transition temperature of PNIPAM within the tested range (0–0.4 M), which encompasses the physiological concentrations. The measurements for adenine and adenosine were limited to concentrations up to 0.1 M due to their limited aqueous solubility. The LCST values of PNIPAM solutions exhibited a decreasing trend as the ATP concentration increased (black squares). Such a trend suggests a clear salting-out behavior.^31^ This contrasts with the anticipated hydrotropic behavior of ATP, which typically involves enhancement of the solubility of substances. Similarly, the presence of TP and AMP also induced a sizable salting-out behavior at the phase transition temperature of PNIPAM. While TP resulted in a substantial monotonic decrease in the phase transition temperature of PNIPAM, similar to the one for ATP, a less dramatic LCST decrease was observed in the presence of AMP. This may suggest that the highly charged and strongly hydrated triphosphate group should dominate the salting-out mechanism. To investigate the distinct behavior of ATP as a proposed hydrotrope, its effect on phase transition temperatures were compared with those of sodium xylene sulfonate (NaXS), a well-established hydrotrope, under similar conditions. In the presence of NaXS, only a marginal change was observed like the ones in adenosine and adenine in the phase transition temperature, even at concentrations as high as 1 M (Figure S2a). Moreover, the LCST was measured at physiological ATP concentrations (starting from 5 mM) in the absence and presence of excess Mg^2+^ and Na^+^ ions, and the same monotonic decreasing trend in the LCST was observed (Figure 1c inset and Figure S2b). The experiment was also repeated with another model macromolecule, poly­(*N,N*-diethyl acrylamide) (PDEA), which shares a similar thermoresponsive behavior with PNIPAM. Despite having slightly different molecular structures—PDEA features diethyl functional groups instead of the isopropyl groups on the amide moiety of PNIPAM—the phase transition results followed the same trend (Figure S3).

In the next set of experiments, ATR-FTIR measurements were performed to investigate the collapsed state of the macromolecule at temperatures above its LCST, at which the macromolecule exists in its collapsed form. This approach provides a molecular-level understanding of the salting-out effect in aqueous systems, in both the presence and absence of ATP or its derivative molecules. This method has previously been employed to elucidate the influence of guanidinium salts to the elastin-like polypeptides.^32^ Figure 2a depicts the scheme of ATR-FTIR measurements, illustrating the sample droplet below and above the LCST, representing the unfolded and collapsed states of the macromolecules, respectively. The dashed lines shown in all of the illustrations show the penetration of attenuated IR light through the diamond ATR crystal. Figure 2b shows the ATR-FTIR measurements of PNIPAM in the absence (0 M) and presence of low (0.05 M) and high (0.2 M) concentrations of ATP at 50 °C (above the LCST). The vibrational fingerprints (amide I (1624 cm^–1^), amide II (1550 cm^–1^), amide III (1450 cm^–1^), and −CH deformation bands (130 – 1400 cm^–1^) indicate the collapsed phase of macromolecules can be observed at all tested conditions (Figure 2b).

**Figure 2:**
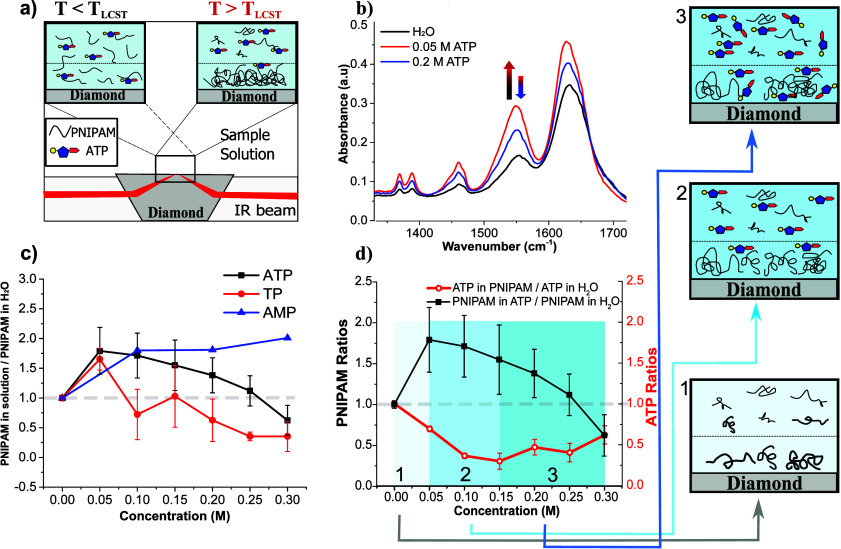
a) The illustration of the PNIPAM in ATP solution droplet below and above the LCST. b) ATR-FTIR spectra of 20 mg/mL PNIPAM in the presence of water (black), 0.05 M ATP (red), and 0.2 M ATP (blue). c) The ratio of PNIPAM in the solutions of ATP, TP, and AMP to PNIPAM in H_2_O. The gray dashed line indicates the region where the PNIPAM concentration in solution equals that in H_2_O (ratio = 1). d) The ratio of PNIPAM in ATP solutions to PNIPAM in H_2_O (black squared) and the peak ratios of ATP solution in PNIPAM to those in H_2_O (hollow red circles). A gray dashed line is also included, indicating where the ratio equals 1.

In Figure 2b, a notable increase in PNIPAM vibrational bands is observed in the presence of 0.05 M ATP compared to PNIPAM solution with no additives (0 M ATP), as clearly seen in the intensity of the amide bands. This result aligns with the salting-out influence exhibited by ATP in the LCST measurements. Such an increase in the intensity suggests a more well-packed collapsed macromolecules on the ATR crystal, which occurs due to the limited penetration depth of the IR light.^32^ However, as the ATP concentration is further increased to 0.2 M, the PNIPAM vibrational band intensities decrease, and thus, a less effective salting-out effect is observed. While this intriguing observation of PNIPAM aggregation persists beyond the concentration of ∼0.05 M, still signifying a salting-out effect, an apparent additional salting-in effect should be at play with increasing ATP concentration, especially above 0.15 M. To clearly illustrate such salting-out action, Figure 2c illustrates the ratio of the aggregation of PNIPAM in ATP solutions with respect to PNIPAM aggregation in the absence of ATP as a function of cosolute concentrations. Here, the ratio is 1 when PNIPAM aggregation in ATP solution is the same as in the no additive sample (in water) (gray dashed line). At 0.05 M ATP, the ratio reaches the maximum value, implying enhanced PNIPAM aggregation. As the ATP concentration increases beyond this point, the curve starts to decrease, suggesting that higher ATP concentrations result in less dense (or fewer) PNIPAM aggregates. A similar trend is observed in the PNIPAM aggregation as a function of TP concentration, although with overall less aggregation than in the presence of ATP. In contrast to both ATP and TP, the aggregation of PNIPAM with AMP only increases without any subsequent reduction, exhibiting an expected trend for a pure salting-out action.

In addition to investigating the collapsed state of the macromolecule, the role of ATP in the collapsed phase of PNIPAM was also examined. To explore the amount of ATP in the PNIPAM aggregates, the P–O stretching peak at 1071 cm^–1^ of ATP in PNIPAM with respect to the ATP solution in the absence of PNIPAM was monitored. The ratio of ATP is shown in Figure 2d (hollow red circles), together with the ratio of PNIPAM data from Figure 2c (black squares) as a function of ATP concentrations. As shown in Figure 2c, the ratio of PNIPAM in ATP compared to H_2_O (depicted as black squares) starts to decrease after reaching a maximum at an ATP concentration of 0.05 M. In sharp contrast to PNIPAM data, the ATP amount in the PNIPAM aggregates significantly decreases up to 0.15 M, after which it begins to slightly increase again. This suggests that as the macromolecules collapse, ATP remains mainly excluded from the macromolecule aggregation. The behavior of reaching a minimum value for ATP is similar to the behavior of Na_2_SO_4_ with elastin-like polypeptides.^32^ Such a behavior is reminiscent of the excluded volume effect dominated by salting-out. It suggests that up to medium ATP concentrations, i.e., ∼0.15 M, the salting-out of the macromolecule is primarily driven by an excluded volume effect. However, above this concentration, the ATP influence shifts to a more modest ATP exclusion. The sketch boxes 1, 2, and 3 illustrate the polymer and ATP in the collapsed state at different concentration regimes of panel d.

Aiming to provide site-specific interaction information between the osmolytes and macromolecules, ^1^H NMR titrations of PNIPAM as a function of ATP, TP, and AMP concentrations were performed (see SI and Figure S4). Only linear chemical shifts were observed for all the PNIPAM hydrogens. Linear chemical shifts for all macromolecular molecular sites have been shown as an indication for the absence of any apparent strong binding interactions.^33–35^ As such, our NMR data also suggest an apparent nonbinding interaction between ATP and the macromolecule. The same noninteracting nature also extends to TP and AMP. In the absence of ATP binding sites and charged functional groups, the baseline interaction for neutral macromolecules is apparently a pure salting-out effect.

To further elucidate the noninteracting nature of ATP with PNIPAM and to investigate the underlying mechanisms responsible for the diminished salting-out effect at elevated ATP concentrations (i.e., 0.15–0.4 M) observed via ATR-FTIR spectroscopy, we performed atomistic molecular dynamics (MD) simulations of single-chain PNIPAM in explicit water and ATP at four representative concentrations. First, we explored the dynamics of 50-mer polymer PNIPAM collapse via running simulations above the LCST (310 K). The simulation was initiated with the polymer in open conformation as shown in Figure 3a, in which the ATP and counterions are homogeneously distributed in the simulation box (see SI figure S8 for initial snapshots). The macromolecule collapses within all tested conditions in the first 100 ns, as can be seen from the radius of gyration (*R*_g_) reduction and stabilization as a function of time (see Figure 3a and Figure S8). In all tested conditions, PNIPAM collapses in the absence and presence of ATP. At the highest concentration tested, PNIPAM seems to have a half-collapsed/half extended state. The distribution of chain sizes for each ATP concentration (i.e., violin plots in Figure 3b) show how ATP can affect size fluctuations in macromolecular aggregation following equilibration (i.e., beyond 100 ns). As compared to the null ATP case, the size distribution is consistently broader in ATP-containing solutions and becomes a maximum at 0.3 M ATP. At this concentration, the single-chain polymer is more swollen, as also evidenced by its increasing radius of gyration (*R*_g_). Figure 3c shows representative snapshots of ATP solutions containing aggregated PNIPAM, captured after equilibrium (see the initial simulation box in Figure S8).

**Figure 3:**
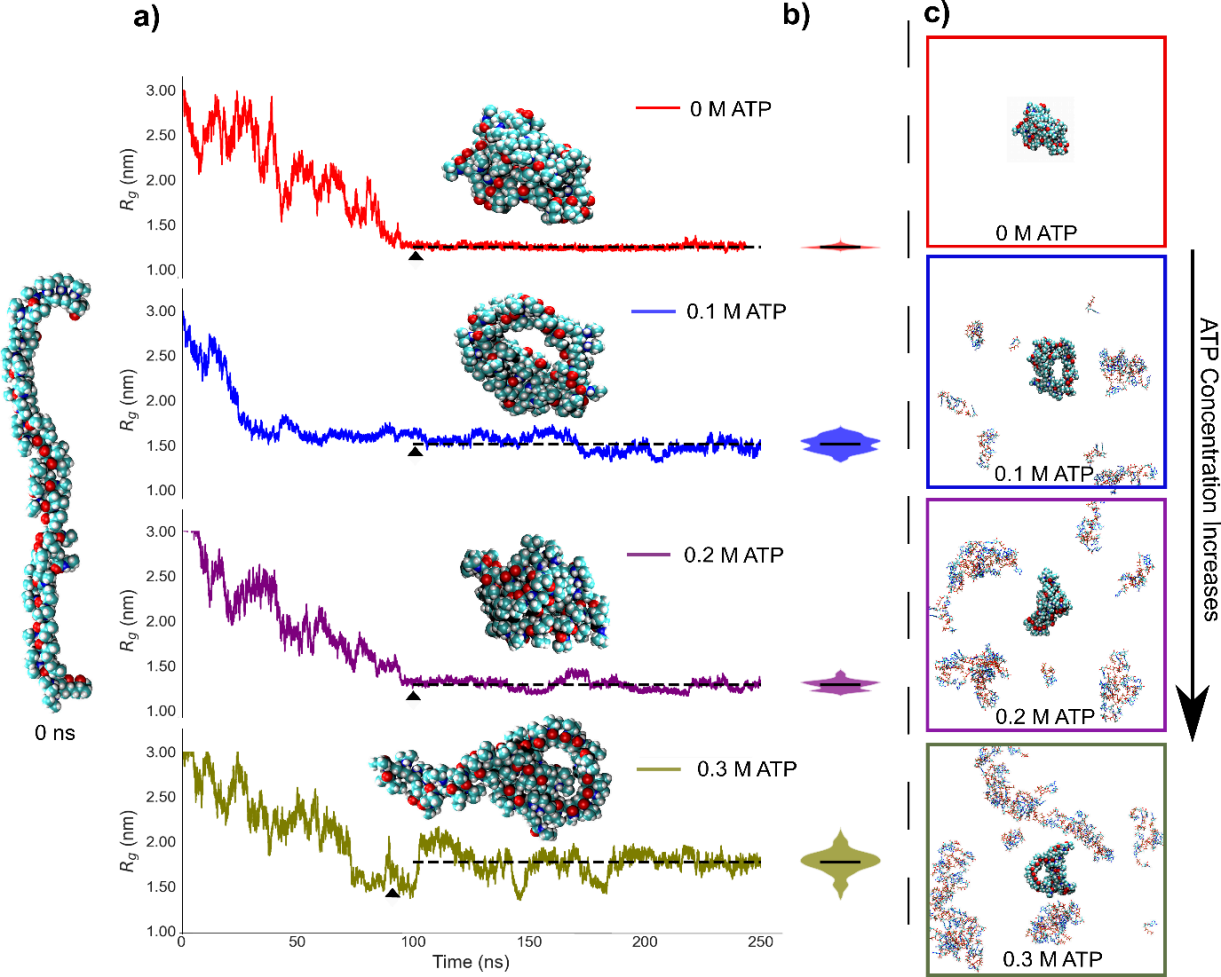
a) The time-traces of end-to-end distance (*R*_g_) for a 50-mer single-chain PNIPAM macromolecule at 310 K, above the phase transition temperature. The total simulation period is 250 ns. A representative simulation snapshot of the PNIPAM chain is shown for each ATP concentration (0 M (red), 0.1 M (blue), 0.2 M (purple), and 0.3 M (yellow) ATP solutions.) The initial configuration of the polymer at the beginning of the simulation is included on the left and labeled as 0 ns. b) Violin plots depict the postequilibration *R*_g_ distributions (calculated at >100 ns). c) The representative snapshots of the simulation box that contain the ATP and the polymer. The water molecules are removed for visual clarity. The time points at which the snapshots were taken are indicated with black arrows on the *R*_g_ plot.

In all simulations, ATP molecules are clearly excluded from the PNIPAM surface, indicating a lack of a statistically significant interaction between ATP and the polymer, corroborating the NMR findings. Notably, ATP molecules exhibit a tendency to form finite-sized clusters, with aggregation becoming more pronounced as the ATP concentration increases. At 0.3 M ATP, all of the ATP molecules coalesce into a few large clusters. Note that it has been reported that ATP shows overpresented aggregation in MD simulations, which can be counteracted by charge scaling of the phosphate moieties of ATP.^11,36^ Importantly, neither individual ATP molecules nor these clusters interact with the macromolecule. This is in fact an interesting result; the noninteracting nature of ATP and macromolecule is completely in line with ATR-FTIR and NMR results (see Figure 2 and Figure S4).

In the last set of experiments, the hydration shell vibrational structure of ATP molecules as a function of concentration was monitored via hydration shell spectroscopy to further investigate the aggregation behavior of ATP molecules experimentally. These measurements were utilized to incorporate characteristics of both the solute’s vibrational bands and its induced perturbations of water OH stretch vibrations. That has been performed on numerous small molecules in aqueous medium.^37–39^ Figure 4 displays the normalized hydration shell spectra of ATP solutions for a concentration range of 0.05–0.4 M, achieved by employing multivariate curve resolution algorithm to pure Raman spectra of the solute and water (see details in SI). With increasing ATP concentration, the normalized hydration spectra gradually alter in the range of 3200–3700 cm^–1^, corresponding to the OH stretch bands. All measured concentrations of ATP exhibit the same spectral features in the CH stretch vibrational region (2870–3130 cm^–1^). Moreover, there are mainly 2 water bands in the hydration spectra, ∼3250 cm^–1^ is referred as more-ordered, and 3430 cm^–1^ corresponds to less-ordered water structure.^40^ The hydration shell spectra provide experimental evidence for water structure changes in the hydration shell of ATP. The normalized intensity of the OH band gradually decreases as a function of ATP concentration. This effect is demonstrated clearly by taking the ratio of the intensity of hydration shell spectra of the OH vibrations (∼3430 cm^–1^) and the CH stretch vibration (∼2950 cm^–1^), shown in the Figure 4 inset. A surprising 59% decrease in the amount of hydrating water molecule decrease per ATP was observed at 0.4 M with respect to 0.05 M of ATP solution. Such concentration-dependent variation reflects a clear change in the hydration characteristics of ATP that should be due to ATP molecules self-assembling into small aggregates, as supported by the MD simulation results. Moreover, the hydration water structure (OH band) also alters slightly but monotonically with an increasing concentration of ATP (Figure S7). Note that the degree of dehydration (59%) is limited, which supports the idea of only small ATP cluster formation. Thus, the experimental results are also in line with the recent charge scaling discussions for MD simulations of ATP molecules.^11,36^

**Figure 4:**
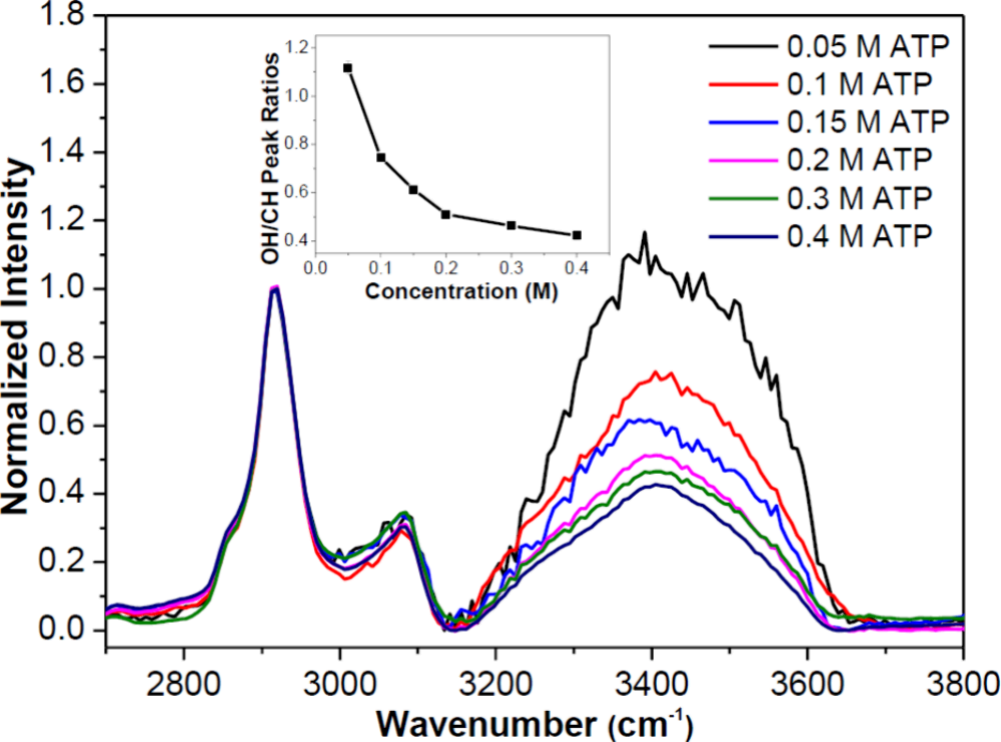
Hydration shell spectra of ATP solutions without macromolecules in concentration range of 0.05–0.4 M. The inset shows the intensity ratio of the OH vibrational band (∼3430 cm^–1^) to the CH stretch vibrational band (∼2950 cm^–1^).

The MD simulation and hydration shell spectroscopy results provide an explanation for the observed diminishing salting-out action of ATP at elevated concentrations. The aggregation of ATP at higher concentrations could potentially be responsible for the disruption of the collapsed phase of PNIPAM by encroaching upon PNIPAM’s spatial arrangement and compelling it into smaller fragments.

ATP is known for its binding affinity to RNA recognition motifs within RNA binding proteins and RGG low-complexity domains (LCDs) present in intrinsically disordered proteins.^19,41–44^ However, ATP interactions extend beyond the binding sites for nucleic acids on proteins. A proteome-wide study revealed that roughly a quarter of the insoluble proteome in human Jurkat cells is solubilized by ATP.^17^ Notably, only a small fraction of the solubilized proteins is known to bind to ATP, while the majority consists of proteins containing intrinsically disordered regions abundant in charged amino acids. The HSQC-NMR analysis has identified the binding to an arginine-enriched IDR region of TDP-43, yet no direct binding has been observed for the arginine-rich globular eye lens protein γS-Crystallin,^15^ which suggests that flexibility or disorder is an enabling factor in determining the binding propensity. However, this does not imply that binding does not take place in proteins that are natively folded. Nishizawa et al.^45^ identified multiple ATP binding sites using NMR on ubiquitin and ubiquitin domain-associated receptor p62 (UBA) within flexible protein regions enriched in charged and hydrophobic residues. In other words, intrinsic disorder and surface dynamics appear to be “enablers” of ATP binding. Ou et al. recently showed by MD simulation and spectroscopy that ATP clusters on folded lysozyme or ubiquitin can dramatically stabilize them, but even in those cases, the binding is highly nonspecific and targets the most flexible, solvated loops.^46^ Sarkar et al. show that ATP binding unfolds a folded Trp-cage miniprotein and extends the disordered Aβ40 peptide via weak hydrophobic and electrostatic contacts.^21^ According to ^1^H–^15^N HSQC experiments, changes in amide chemical shifts were observed in resonances associated with the main hydrophobic surface of Ub (T9, I44, H68, and V70).^45^ ATP associates with Ub by forming noncovalent weak interactions primarily involving hydrophobic and charged amino acids, particularly those around the I44 hydrophobic patch, including V70. Specifically, binding occurs with the C-terminal tails for both proteins and the loop region of ubiquitin.^45^

In the case of positively charged proteins such as lysozyme and the IDP histatin-5, low concentrations of triphosphate have the opposite effect of inducing protein precipitation.^28,29^ Likewise, ATP has been demonstrated to enhance fibril formation in various basic IDPs,^26^ along with an insulin fragment conjugated to octalysine.^27^ In these scenarios, the ion-specific effects were attributed to polyphosphate, which creates ion pairs between the charged groups of proteins. Given that ATP binding to valine residue (V70) was demonstrated on Ubiquitin, one might expect an interaction between ATP and the model macromolecule PNIPAM, which contains an isopropyl group in its structure resembling valine. Additionally, since PNIPAM possesses a random-coil structure akin to intrinsically disordered proteins (IDPs), one would expect hydrotropic action. Contrary to the expectations, a clear salting-out effect of ATP with no apparent specific binding was observed between ATP (and ATP derivatives) and the model macromolecules. This implies that the actual hydrotropic action is not just a result of open chain structure or pure hydrophobic nature of the macromolecule. ATP action on hydrophobic macromolecules has not been studied before with an experimental approach. MD simulations by Sarkar *et al.* demonstrated weak interaction between ATP and 32 unit coarse-grained hydrophobic chain.^20^ ATP induced salting-out of caffeine^47^ and Disperse Red 13,^11^ while it only weakly solubilized pyrene^48^ via π–π stacking between ATP’s adenine ring and the pyrene aromatic core. Herein, for neutral PNIPAM and PDEA macromolecules, a clear salting-out action of ATP was demonstrated that is dominated by an excluded volume effect mechanism. Such an effect is observable even at physiologically relevant concentrations as low as 5 mM (Figure 1c inset). ATP beyond 0.05 M gradually shows small cluster formation that leads to disruption of the collapsed phase of PNIPAM and compelling it into smaller fragments. Thus, at elevated concentrations, ATP can function as an aggregation disruptor.

In summary, we performed a multi-instrumental experimental study of ATP interaction with a model neutral macromolecule in combination with MD simulations. The phase transition temperature measurements indicated a pure salting-out behavior of ATP, TP, and AMP on macromolecules, whereas adenine and adenosine groups did not significantly influence the phase transition of the macromolecules. ATR-FTIR measurements supported the salting-out behavior of ATP, TP, and AMP molecules and revealed a surprising decreasing trend in the salting-out behavior of ATP, and TP at high concentrations. AMP, on the other hand, showed regular salting-out behavior with a monotonic increase in its salting-out influence. ATR-FTIR measurements also revealed that ATP molecules are excluded from the collapsed form of macromolecules, indicating a dominant excluded volume effect. Additionally, the NMR measurements showed no apparent saturable binding interaction between ATP and the macromolecule. The MD simulations confirm that ATP and PNIPAM have weak to no steric interactions within the time scale much longer than the average diffusion time of individual ATP molecules. The simulations also revealed some degree of ATP clustering, specifically at high concentrations. To further explore the ATP clustering, we conducted hydration shell spectroscopy. The spectra show a gradual decreasing trend in the OH bands, which indicates dehydration occurring between the ATP molecules. In other words, there is less water per ATP molecule as the concentration of ATP increases. The formation of small ATP clusters at higher ATP concentrations generates an additional effect, enabling PNIPAM to form smaller aggregates in its collapsed form. Overall, all these findings are interpreted as ATP acting as a stabilizer on neutral macromolecules, proposing a twist to a sole hydrotropic behavior.

## Supplementary Material



## Notes

Additional experimental details, materials and methods, and phase transition temperature data of PNIPAM and PDEA, 1H-NMR with all molecules, hydration shell spectra of ATP, simulation details and additional simulation data and analysis (PDF)
