# Supplementary material for: ATP Can Act as a Stabilizer on Neutral Macromolecules
Source: J Phys Chem Lett. 2025 Oct 7;16(41):10771–7. doi: 10.1021/acs.jpclett.5c02467 (PMC12536438; doi:10.1021/acs.jpclett.5c02467)
Supplement: Supplementary file 1 [file jz5c02467_si_001.pdf]

Supporting Information

ATP Can Act as a Stabilizer for Neutral Macromolecules

Cansin Ayvaz[1], Yaren S. Ozdogan[1], Dilsad S. Peker[1], Aykut Erbas[2*], Halil I. Okur[1,2*]

[1] Department of Chemistry, Faculty of Science, Bilkent University, 06800 Ankara Turkey.

[2] National Nanotechnology Research Center (UNAM), Bilkent University, 06800 Ankara, Turkey

Contents

S1 Experimental Details

S2 Simulation Details

S3 Supporting Experiments

S1. Experimental Details

Materials

The chemicals utilized in this study are listed below. Poly(*N*-isopropyl acrylamide) (PNIPAM) (Polymer Source, Inc., Mw: 118.500 g/mol), poly(*N*-isopropyl acrylamide) (PNIPAM) (Sigma Aldrich, Mw: 85.000 g/mol), poly(*N*, *N*- diethyl acrylamide) (PDEA) (Polymer Source, Inc., Mw: 55.000 g/mol), adenosine (Sigma Aldrich, 99% purity), adenine (Sigma Aldrich, 99% purity), sodium triphosphate pentabasic (Sigma Aldrich, 98% purity), adenosine 5'-triphosphate disodium (ATP) (Sigma Aldrich, 99% purity), adenosine 5'-monophosphate sodium salt (Sigma Aldrich, 99 % purity), sodium xylene sulfonate ($NaXSO_4$) (Sigma Aldrich, mixture of isomers, 40 wt. % in H_2O), H_2O (Millipore Nanopure system, 18.2 M cm), Deuterium Oxide (D_2O) (Eurisotop, 99.90% D) were used without any further purification.

Lower Critical Solution Temperature (LCST) Measurements

Phase transition measurements of 5 mg/mL PNIPAM and PDEA were made with an OptiMelt MPA 100 instrument (Stanford Research Systems, USA). Each sample was measured at least with 3 capillary tubes, each containing a minimum of 10 µL of desired solution. The capillary tubes had 1.2-1.5 x 80 mm dimensions and were purchased from Paul Marienfeld GmbH & Co KG. The temperature is controlled and ramped with at a rate of 1 °C/min. The phase transition temperature (also known as lower critical solution temperature - LCST -) were taken as the onset of the rise in detected scattering, the intersection of the linearized curves before and after the onset (as shown in Figure S1)

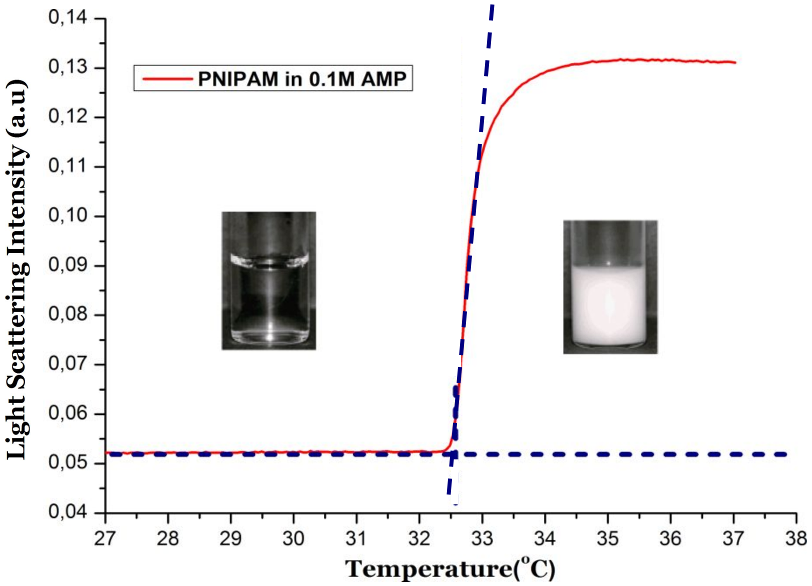

Figure S1: The graph displays the scattering light intensity (a.u.) plotted against temperature (°C) for PNIPAM in 0.02 M AMP. The image on the left corresponds to the soluble state of PNIPAM below the LCST, while the image on the right represents the collapsed state of PNIPAM above the LCST. Dashed lines are placed on both axes of the graph to indicate the starting point of the light intensity change over the temperature range.

ATR-FTIR Measurements

A Bruker ALPHA II compact FTIR spectrometer (Bruker, USA) with an ATR attachment was used to measure all IR spectra. All the spectra were taken at 2 cm^{-1} resolution and both the sample and background consist of 32 scans each. All spectra were taken at 50 °C. The samples were analyzed in the following sequence: pure H_2O, polymer (PNIPAM or PDEA) in H_2O, ATP/TP/AMP in H_2O at concentrations ranging from 0.01 to 0.5 M, and a sample containing both polymer (PNIPAM and PDEA) and ATP/TP/AMP aqueous solution. For each measurement, 50 μL of solution was used, and spectra were captured at four time points: immediately after placing on the ATR crystal, and after 3 minutes, 5 minutes, and 10 minutes. The measurements after 5 minutes are presented in this article, yet the same trends can be seen for all time interval data.

Nuclear Magnetic Resonance (NMR)

^{1}H-NMR measurements were utilized using a Bruker AVANCE III 400 MHz spectrometer. All NMR samples were measured at room temperature, which is below the phase transition temperature of PNIPAM in all measured samples. The spectra were calibrated with an external reference, sodium 2,2-dimethyl-2-silapentane-5-sulfonate (DSS). The PNIPAM samples were

placed in the outer concentric tube, while the DSS was in the inner tube. Therefore, the DSS control was never in contact with the PNIPAM solutions. Data processing was carried out using the TopSpin software from Bruker and the MestReNova software from Santiago de Compostela, Spain.

Hydration Shell Spectroscopy (Multivariate Curve Resolution Raman Spectroscopy)

Using a Low Noise Continuous Wave (CW) Diode-Pumped Solid-State (DPSS) Laser (Ventus 532) at 532 nm at 50 mW with 10 % attenuation as the excitation laser source, the samples Raman spectra were measured with a Jobin Yvon Horiba Raman System with an Andor charge-coupled device (CCD) camera. It was employed by a < 0.01 electron/pixel/second dark current response by thermo-electrically cooling the detector to -69 ^{o}C. The laser light is directed to the sample and collected with a 10x objective. The collected light was directed to 600 g/mm grating and finally measured by a 1024 x 256 pixels CCD camera. Unless specified otherwise, spectra were obtained using 5 minutes integration period. All the measurements were performed at ambient temperatures. The Raman signal of aqueous solutions contains the intrinsic chemical information regarding the solvent, solute, and the hydration shell of the solvent around the solute molecule. The backscattered Raman light from the middle of the aqueous solution container in spectroscopic quartz cuvettes was measured. A self-modeling Multivariate Curve Resolution (sMCR) algorithm that was previously optimized self-modeling Multivariate Curve Resolution (sMCR) utilized in Raman-MCR measurements to reach the solute correlated spectrum has been employed in this study[1,2].

S2 Simulation Details

Molecular Dynamics Simulation (MD)

The molecular dynamics (MD) simulations were carried out with GROMACS[3] simulation package version 2021.5. The simulations of fully atomistic models of uncharged PNIPAM chains consisting of 50 monomers were performed in a cubic simulation cell which was filled with ATP and water molecules. The dimensions of the cubic box were 15.38 nm in each direction. Various simulations were performed, each involving the same PNIPAM chain with changing ATP concentration. The concentrations of ATP investigated were 0 M, 0.1 M, 0.2 M, and 0.3 M. The charge of ATP is neutralized by the addition of Na^+ ions.

All molecular dynamics simulations were conducted using the CHARMM36[4] family of force fields, with topologies converted to GROMACS format via the CHARMM-GUI FF-Converter. Parameters for the PNIPAM polymer were generated with the CHARMM-GUI Polymer Builder[5], ensuring compatibility with CHARMM36 polymer definitions. ATP parameters were obtained from the CHARMM36 nucleotide parameter set available through CHARMM-GUI [6]. Water molecules were described with the SPC/E[7] model, and sodium/chloride ions with the standard CHARMM36 ion parameters. This setup ensured a consistent CHARMM36-based representation across all system components.

The system underwent energy minimization using the steepest-descent algorithm before the final Molecular Dynamics (MD) simulation. The MD simulations lasted for 250 ns and were conducted in an NPT ensemble at a temperature of 310 K, which is above the Lower Critical Solution Temperature (LCST) of PNIPAM in pure water. In the simulations, the temperature was controlled using a velocity rescale thermostat with a time constant of 0.1 ps, while the pressure was maintained at an equilibrium value of 1 bar through the use of a Parrinello-Rahman barostat with a time constant of 2.0 ps. To enhance statistical robustness, each simulation was replicated twice. Periodic boundary conditions are applied in all three dimensions. Time propagation is accomplished using a leapfrog integrator with a time step of 2 fs. For van der Waals and Coulomb interactions, a cutoff of 1.2 nm is utilized. Long-range electrostatic interactions are treated using the particle-mesh Ewald (PME) method with a grid spacing of 0.16 nm. The LINCS32 algorithm is used to constrain the bonds involving hydrogen atoms and also the bonds and angles of the water molecules. For energy and pressure, long-range dispersion corrections have been used. The trajectories obtained for all systems were analyzed using Visual Molecular Dynamics (VMD)[8].

S3 Supporting Experiments

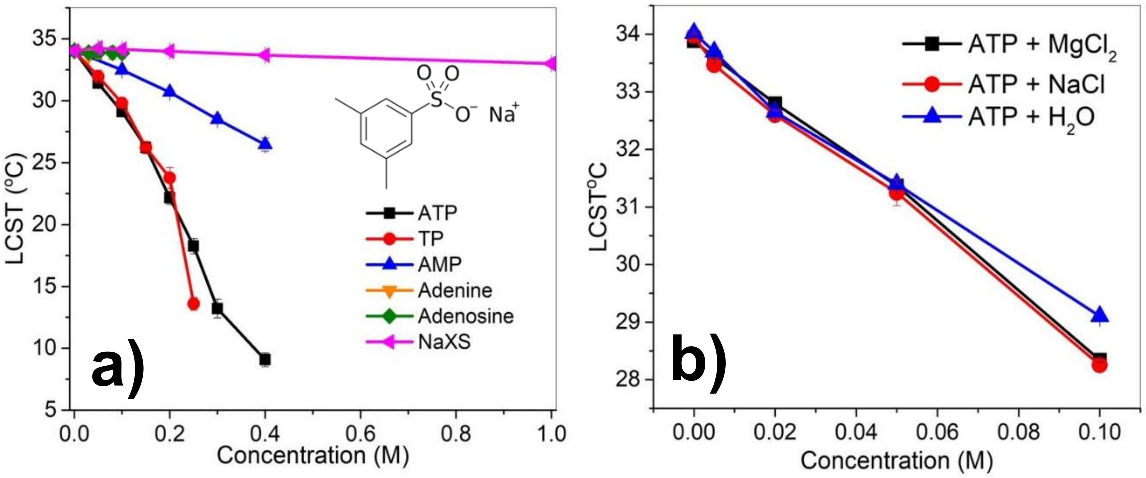

Figure S2: a) The LCST of 5 mg/mL PNIPAM as a function of ATP, TP, AMP, adenine, adenosine, and Sodium xylene sulfonate (NaXS) concentrations (M). Error bars indicate the standard deviation of data. The solid lines are a guide to the eye. The inset shows the structure of sodium xylene sulfonate (NaXS). b) The LCST of 5 mg/mL PNIPAM in the presence of ATP in $MgCl_2$, ATP in NaCl, and ATP in aqueous solutions, as a function of ATP concentrations (M). The additional salt was kept at 10 mM for NaCl and $MgCl_2$. Error bars indicate the standard deviation of data. The solid lines are a guide to the eye.

In Figure S2a, LCST measurements of PNIPAM were conducted in the presence of a well-known chemical hydrotrope, NaXS, the structure of which can be seen in the inset of Figure S2a. For a comprehensive comparison of the effects on the LCST of PNIPAM, all the results of LCST measurements in ATP, TP, and AMP solutions were plotted together with the NaXS, and other sets of data are plotted together. As also shown in Figure 1c, ATP, TP, and AMP exhibit salting-out behavior on the phase transition temperature of PNIPAM, while NaXS, a well-known chemical hydrotrope, follows a linear trend similar to adenine and adenosine but even at higher concentrations. In other words, NaXS does not exhibit any salting-out behavior with PNIPAM macromolecule. Such results along with the literature discussion in the main text demonstrate the influence of ATP on macromolecules is beyond the general expected behavior of an hydrotrope.

In Figure S2b, the impact of the presence of physiologically relevant cations on the phase transition of macromolecules was investigated. The presence of Mg^{2+} ions was proposed to be a key ingredient for its hydrotropic action[9]. To test the influence of Mg^{2+} cations, 10 mM of $MgCl_2$ and 10 mM of NaCl salts were maintained with varying ATP concentrations and the phase

transition of PNIPAM was measured. As can be seen from Figure S2b, the LCST value of PNIPAM show no significant change with the presence of these additional metal chloride salts. Such a result suggests that the observed unexpected salting-out behavior of ATP on the phase behavior of PNIPAM is independent of the presence of other physiological cations, Mg^{2+}, and Na^{+}.

The effect of ATP, adenosine, adenine and triphosphate on the phase transition of PNIPAM is demonstrated clearly. The salting-out behavior of the macromolecule in the presence of ATP and triphosphate have also been tested with another macromolecule i.e. poly(*N,N*-diethyl acrylamide) (PDEA). Figure S3 demonstrates the phase transition temperature of PDEA as a function of ATP, TP, adenine and adenosine. The structure of PDEA can be seen in the inset. Surprisingly, similar phase transition temperature behavior in the presence of ATP and TP, as well as adenine and adenosine can clearly be seen. The former two yield a clear salt-out whereas the latter two show unaltered LCST values. As such, one can claim that the salting-out behavior of these molecules is not a rare anomaly.

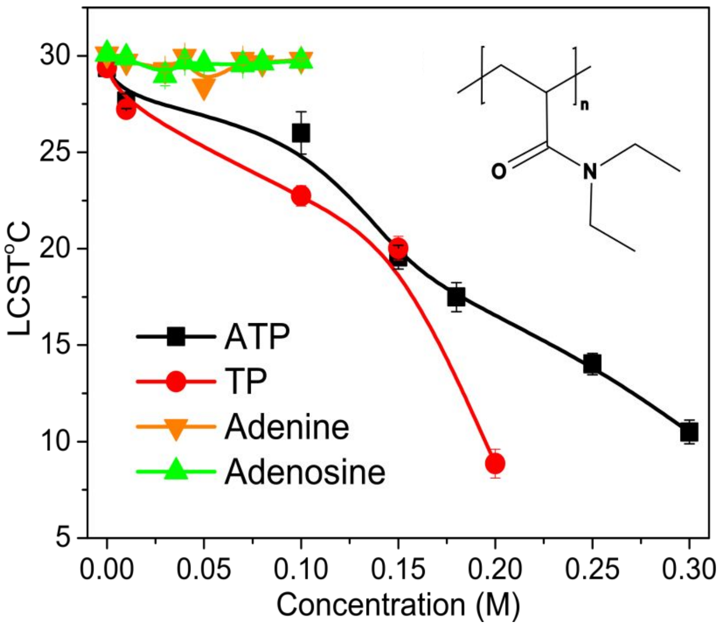

Figure S3: The LCST of 5 mg/mL PDEA as a function of ATP, TP, Adenine, and Adenosine concentrations (M). Error bars indicate the standard deviation of data for many data points, it is within the data points drawn. The solid lines are a guide to the eye. The structure of poly(*N*, *N*-diethyl acrylamide) (PDEA) is shown in the inset.

One may argue that the salting-out effect of ATP can be due to complex ion-macromolecule interactions[10,11]. In order to see if there is any interaction of ATP with PNIPAM macromolecule, ^{1}H-NMR measurements were performed. The ^{1}H-NMR titrations of PNIPAM in deuterated solutions of ATP, TP, and AMP were conducted. ^{1}H-NMR has recently been employed to decipher any binding interactions between small molecules/ions and macromolecules.[12] Figure S4a illustrates the ^{1}H-NMR spectrum of PNIPAM in neat D_2O, that is externally referenced to sodium trimethylsilylpropane sulfonate (DSS) in D_2O. The four major chemical shifts (color & symbol-coded) on the spectrum correspond to distinct protons of the PNIPAM macromolecule, identified with red circle, yellow square, green star, and blue triangle. The lowest chemical shift signal, denoted with a green start is positioned at ~1.15 ppm, corresponding to the six i-Pr terminal methyl hydrogens. The following chemical shift, indicated by a yellow square at approximately 1.58 ppm, corresponds to the backbone methylene ($-CH_2$) group, which is positioned relatively far from the electronegative groups. The -CH backbone proton, alpha position to the amide carbonyl (indicated with a red circle), is positioned at around 2.02 ppm because it is relatively closer to the electron-withdrawing carbonyl group. Lastly, the highest chemical shift signal of PNIPAM belongs to the -CH- group adjacent to the amide N-atom (blue triangle) and appears at ~ 3.90 ppm. The measurements are shown in panels b-e of Figure S4, where the change in chemical shift ($\delta\Delta$ ppm) for each proton signal is plotted as a function of ATP, AMP, and TP concentrations. The positions of the protons are labeled using the same color coding as in Figure S4a. All of the chemical shift changes at specific sites of PNIPAM in the presence of ATP, AMP, and TP exhibit a completely linear and monotonic decrease. Note that, since all the groups show linear decreasing shifts without any nonlinear deviation in any specific groups, no apparent binding interaction can be demonstrated.

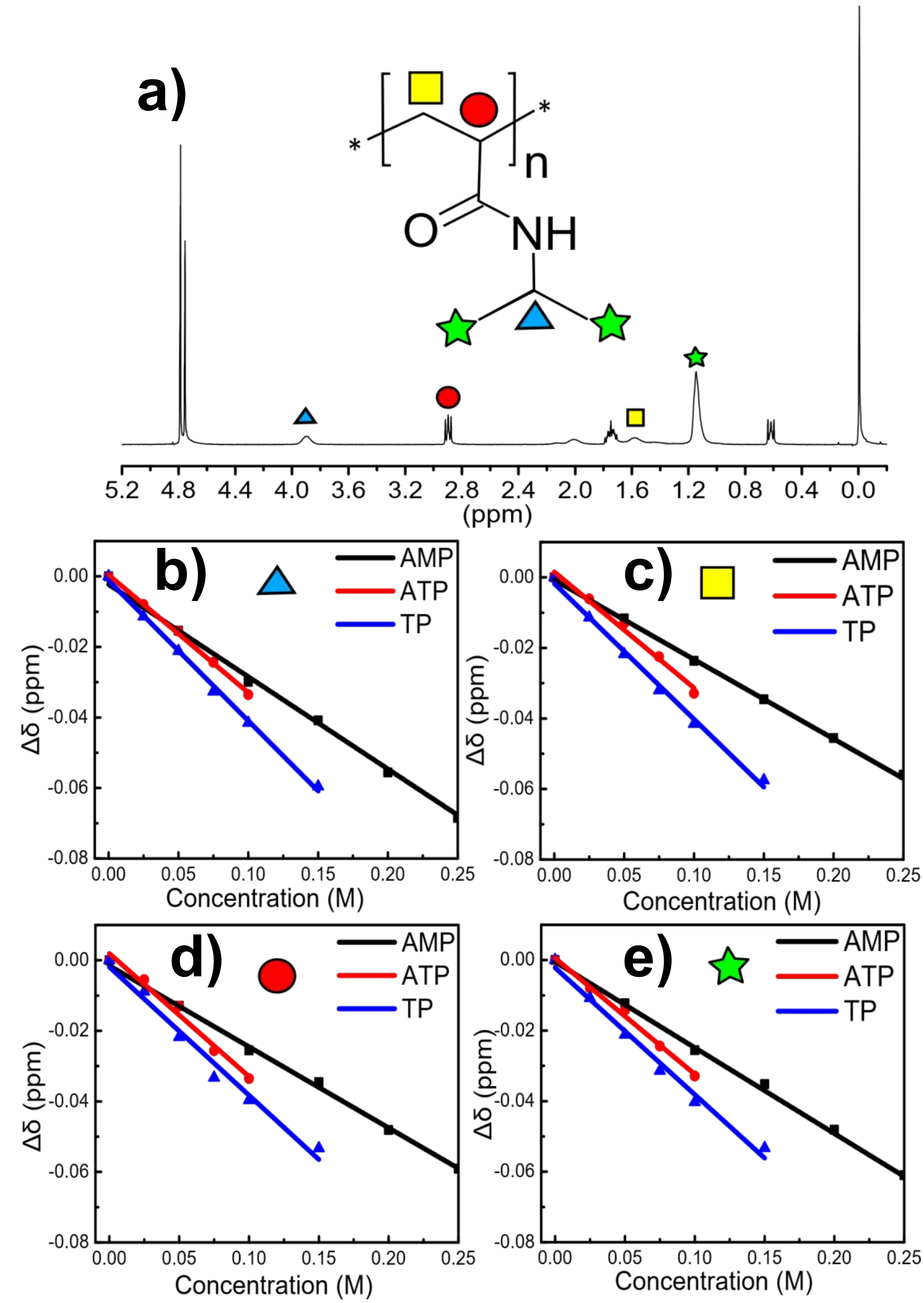

Figure S4: a) ^{1}H-NMR titrations of 3.3 mg/mL PNIPAM in D_2O. The structure of PNIPAM is displayed on the top of the figure, in which each different proton indicated as colored symbols. The four panels (b-e) illustrate the changes in the chemical shifts of each specific site as a function of the concentration of ATP, AMP, and TP. b) (blue) the N-CH group, c) (yellow) the backbone -CH_2- group, d) (red) the backbone -CH- group (alpha position to carbonyl group), e) (green) i-Pr terminal -CH_3 groups.

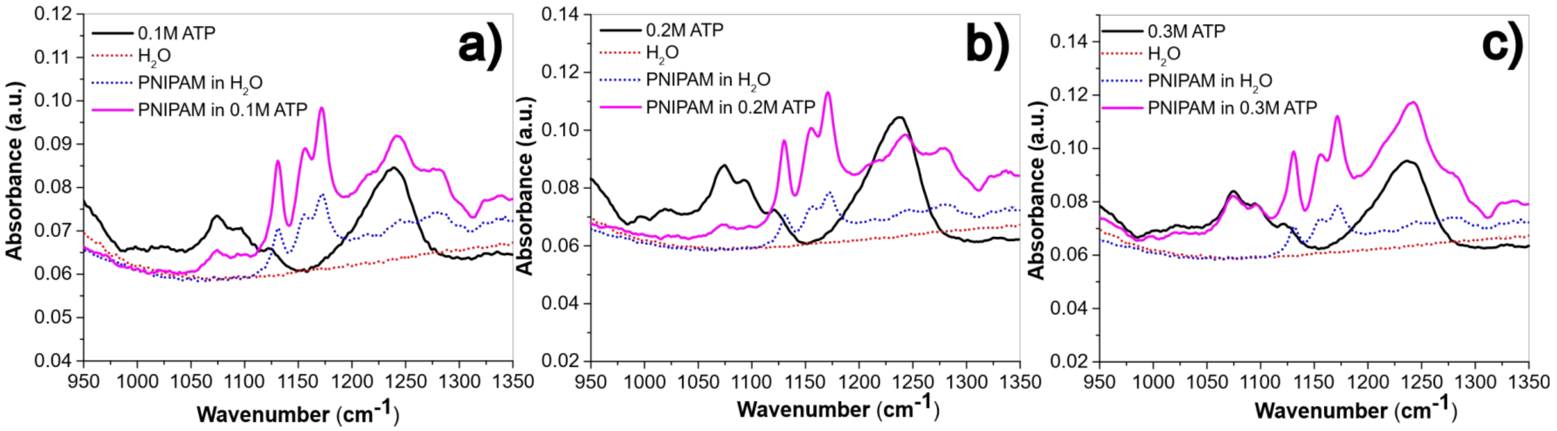

Figure S5: a) ATR-FTIR spectra of water (dashed red), 0.1 M ATP (black), 20 mg/mL PNIPAM in the presence of water (dashed blue), 20 mg/mL PNIPAM in the presence of 0.1 M ATP (pink). B) Same spectra with ATP concentration increased to 0.2 M. c) Same spectra with ATP concentration increased to 0.3 M.

The ratios given in Figure 2c and 2d were calculated by using the spectra of PNIPAM in H_2O, PNIPAM in ATP solution and only ATP solution. A set of such data can be seen in Figure S5.

The hydration spectrum of ATP is achieved by utilizing the sMCR algorithm to the Raman spectra of DI water and 0.4 M ATP solution (Figure S6a). The deconvoluted hydration shell spectrum is shown Figure S6b. Figure S7 shows the normalized hydration shell spectra of ATP at various concentrations. A clear monotonic decrease in intensity of ~ 3500 cm^{-1} band as a function of ATP concentration can clearly be seen where this frequency corresponds to the less-tetrahedrally coordinated water molecules. Such spectral change indicates the native soluble state of ATP at low concentration alters as the concentration increases both the amount of hydration water molecules around an ATP (Figure 4 inset) and the hydration structure (Figure S7) changes.

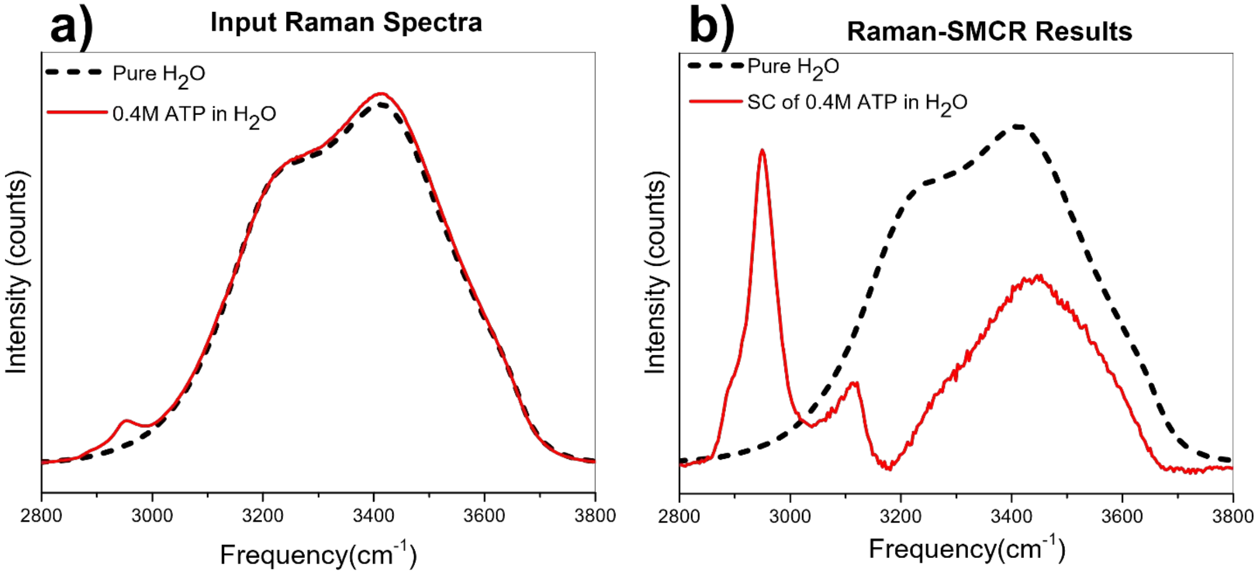

Figure S6: a) Raman spectra of pure water (dashed black) and 0.4 M aqueous ATP solution (red). b) Solute correlated (SC) spectrum (aka hydration shell spectrum) of 0.4 M ATP (solid red) and Raman spectrum of pure water (dashed black). The features in the SC spectrum arise from the vibrational modes of ATP and water molecules influenced by ATP, displaying differences compared to bulk water.

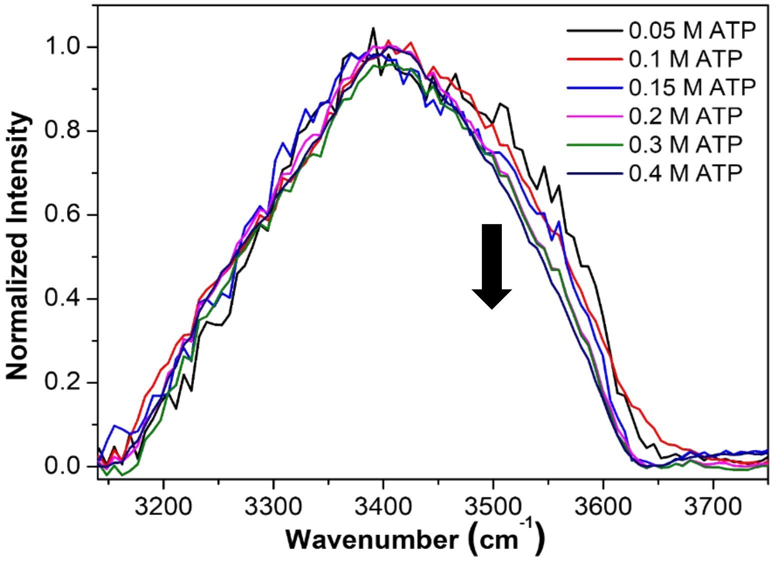

Figure S7: Raman SC spectra of ATP solutions at the concentration range of 0.05-0.4 M after performing counterion subtraction by using Raman multivariate curve resolution analysis. The arrow shows the decreasing intensity of less-tetrahedrally coordinated water molecules in the hydration shells with increasing ATP concentration.

Figure S8a shows the representative initial snapshots from the simulations, displaying the PNIPAM chain in the presence of sodium ions and ATP at concentrations of 0 M, 0.1 M, 0.2 M, and 0.3 M, respectively. Figure S8b shows solvent accessible surface of area of PNIPAM in all tested simulation conditions. From the simulations snapshots one can quantify any binding interactions. In order to quantify the direct interaction of the cosolute molecule (ATP, in our case) with the macromolecular chain in simulations, preferential binding coefficient (Γ) was calculated. Γ is defined as follows:

$$\Gamma = N_{tot}\left(\frac{n_a}{n_w} - \frac{N_{tot}}{N_{wtot}}\right) \quad \text{(Equation S1)}$$

where N_{tot} is the total number of ATP molecules in the simulation box, n_a is the number of bound ATP, n_w is the number of surface bound water molecules, and N_{wtot} is the total number of water molecules in the system. Since no apparent ATP binding was observed, slight negative values were calculated for Γ.

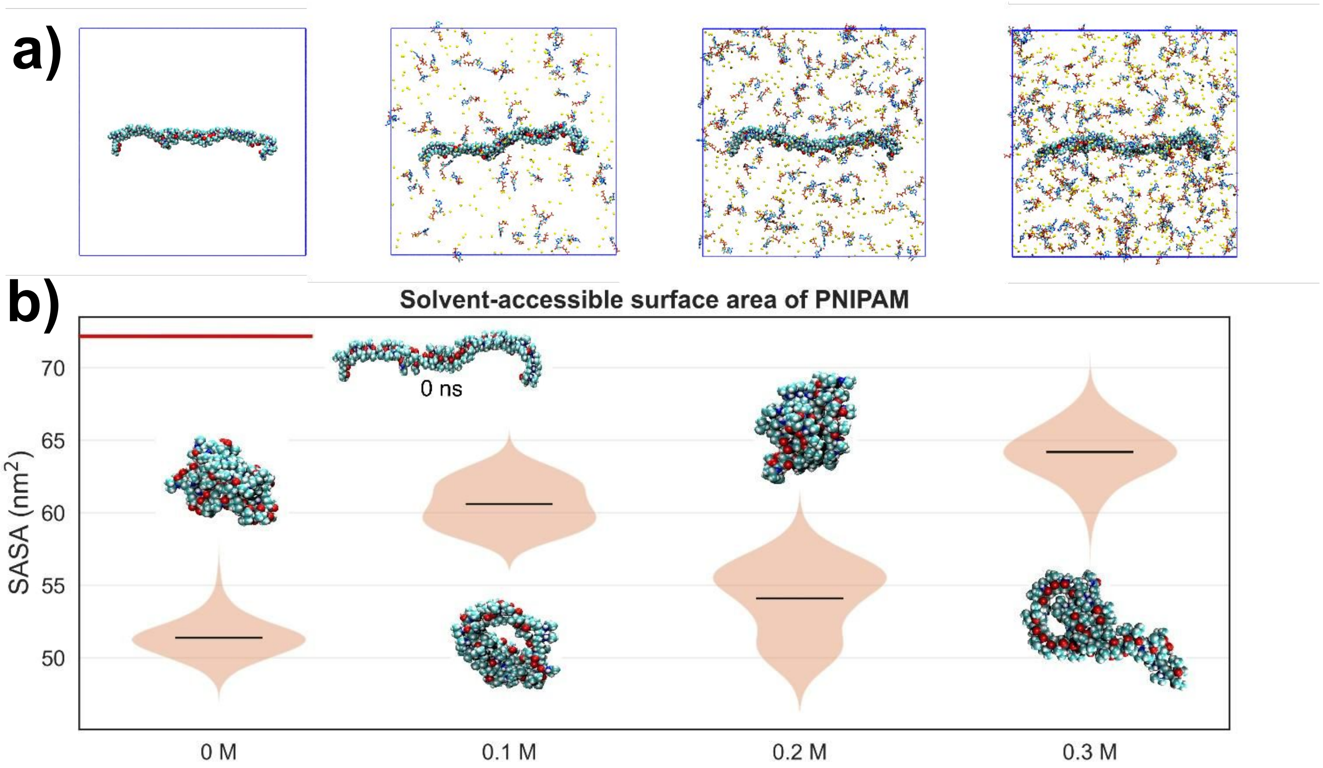

Figure S8: a) Representative initial snapshots from the simulations, displaying the PNIPAM chain in the presence of sodium ions and ATP at concentrations of 0 M, 0.1 M, 0.2 M, and 0.3 M, respectively (from left to right). Water molecules are omitted for clarity due to visual crowding.

b) Solvent accessible surface area (SASA) distributions of PNIPAM at varying ATP concentrations on the x axis. Violin plots represent the SASA values sampled after 100 ns of simulation, when the system has reached equilibrium. For each concentration, the representative PNIPAM snapshots are shown. The red horizontal line denotes the SASA value of the PNIPAM conformation at the beginning of the simulation, and the corresponding snapshot is shown on the right.

References

(1) Davis, J. G.; Gierszal, K. P.; Wang, P.; Ben-Amotz, D. Water Structural Transformation at Molecular Hydrophobic Interfaces. *Nature* 2012, *491* (7425), 582–585. https://doi.org/10.1038/nature11570.

(2) Ben-Amotz, D. Hydration-Shell Vibrational Spectroscopy. *J Am Chem Soc* 2019, *141* (27), 10569–10580. https://doi.org/10.1021/jacs.9b02742.

(3) Abraham, M. J.; Murtola, T.; Schulz, R.; Páll, S.; Smith, J. C.; Hess, B.; Lindahl, E. GROMACS: High Performance Molecular Simulations through Multi-Level Parallelism from Laptops to Supercomputers. *SoftwareX* 2015, *1–2*, 19–25. https://doi.org/10.1016/j.softx.2015.06.001.

(4) Best, R. B.; Zhu, X.; Shim, J.; Lopes, P. E. M.; Mittal, J.; Feig, M.; MacKerell, A. D. Optimization of the Additive CHARMM All-Atom Protein Force Field Targeting Improved Sampling of the Backbone φ, ψ and Side-Chain χ 1 and χ 2 Dihedral Angles. *J Chem Theory Comput* 2012, *8* (9), 3257–3273. https://doi.org/10.1021/ct300400x.

(5) Choi, Y. K.; Park, S.-J.; Park, S.; Kim, S.; Kern, N. R.; Lee, J.; Im, W. CHARMM-GUI Polymer Builder for Modeling and Simulation of Synthetic Polymers. *J Chem Theory Comput* 2021, *17* (4), 2431–2443. https://doi.org/10.1021/acs.jctc.1c00169.

(6) Jo, S.; Kim, T.; Iyer, V. G.; Im, W. CHARMM-GUI: A Web-based Graphical User Interface for CHARMM. *J Comput Chem* 2008, *29* (11), 1859–1865. https://doi.org/10.1002/jcc.20945.

(7) Berendsen, H. J. C.; Grigera, J. R.; Straatsma, T. P. The Missing Term in Effective Pair Potentials. *J Phys Chem* 1987, *91* (24), 6269–6271. https://doi.org/10.1021/j100308a038.

(8) Humphrey, W.; Dalke, A.; Schulten, K. VMD: Visual Molecular Dynamics. *J Mol Graph* 1996, *14* (1), 33–38. https://doi.org/10.1016/0263-7855(96)00018-5.

(9) Patel, A.; Malinovska, L.; Saha, S.; Wang, J.; Alberti, S.; Krishnan, Y.; Hyman, A. A. ATP as a Biological Hydrotrope. *Science (1979)* 2017, *356* (6339), 753–756. https://doi.org/10.1126/science.aaf6846.

(10) Heyda, J.; Okur, H. I.; Hladílková, J.; Rembert, K. B.; Hunn, W.; Yang, T.; Dzubiella, J.; Jungwirth, P.; Cremer, P. S. Guanidinium Can Both Cause and Prevent the Hydrophobic Collapse of Biomacromolecules. *J Am Chem Soc* 2017, *139* (2), 863–870. https://doi.org/10.1021/jacs.6b11082.
(11) Bruce, E. E.; Bui, P. T.; Rogers, B. A.; Cremer, P. S.; van der Vegt, N. F. A. Nonadditive Ion Effects Drive Both Collapse and Swelling of Thermoresponsive Polymers in Water. *J Am Chem Soc* 2019, *141* (16), 6609–6616. https://doi.org/10.1021/jacs.9b00295.
(12) Rogers, B. A.; Okur, H. I.; Yan, C.; Yang, T.; Heyda, J.; Cremer, P. S. Weakly Hydrated Anions Bind to Polymers but Not Monomers in Aqueous Solutions. *Nat Chem* 2022, *14* (1), 40–45. https://doi.org/10.1038/s41557-021-00805-z.
